# Short Forms of Wechsler Scales Assessing the Intellectually Gifted Children Using Simulation Data

**DOI:** 10.3389/fpsyg.2018.00830

**Published:** 2018-05-28

**Authors:** Alexandre Aubry, Béatrice Bourdin

**Affiliations:** CRP-CPO EA 7273, Université de Picardie Jules Verne, Amiens, France

**Keywords:** intelligence, brief assessment, gifted children, short-form, screening tools

## Abstract

Intellectual giftedness is usually defined in terms of having a very high Intellectual Quotient (IQ). The intellectual capacity is assessed by a standardized test such as the Wechsler Intelligence Scale for Children (WISC). However, the identification of intellectually gifted children (IGC) often remains time-consuming. A short-form WISC can be used as a screening instrument. The practitioners and researchers in this field can then make a more in-depth evaluation of the IGC's cognitive and socioemotional characteristics if needed. The aim of our study is thus to determine the best short tests, in terms of their psychometric qualities, for the identification of IGC. The current study is composed of three-step analyses. Firstly, we created nine IQs short forms (IQ_SF_) with 2-subtests, and nine IQ_SF_ with 4-subtests from the WISC-IV (Wechsler, 2005). Secondly, we estimated psychometric parameters (i.e., reliability and validity) from empirical and simulated dataset with WISC-IV. The difference in the estimation of psychometric qualities of each IQ_SF_ from the simulated data is very close to those derived from empirical data. We thus selected the three best IQ_SF_ based on these psychometrics parameters estimated from simulated datasets. For each selected short form of the WISC-IV, we estimated the screening quality in our sample of IGC. Thirdly, we created IQ_SF_ with 2- and 4-subtests from the WISC-V (Wechsler, 2016) with simulated dataset. We then highlighted the three best short forms of WISC-V based on the estimated psychometric parameters. The results are interpreted in terms of validity, reliability and screening quality of IGC. In spite of the important changes in the WISC-V, our findings show that the 2-subtest form, *Similitaries* + *Matrix Reasoning*, and 4-subtest form, *Similitaries* + *Vocabulary* + *Matrix Reasoning* + *Block Design*, are the most efficient to identify the IGC at the two recent versions of Wechsler scales. Finally, we discuss the advantages and drawbacks of a brief assessment of intellectual aptitudes for the identification of the IGC.

## Introduction

In the sphere of education, the rapid and reliable evaluation of a child's global intellectual capacity is important for an efficient identification of intellectually gifted children (IGC). Indeed, such evaluation contributes in proposing specific educational programs (e.g., accelerated or enrichment programs). In this context, a short evaluation can initially be used to assess a child's intellectual giftedness. It can then serve to determine whether a more in-depth evaluation of the child's cognitive and socioemotional characteristics is needed.

The importance and usefulness of cognitive ability assessments for the identification of IGC have long been recognized by the scientific community as a means of facilitating the integration of IGC in specialized school programs (Simpson et al., 2002; Pierson et al., 2012). Furthermore, special education such as enrichment education has proved to impact positively on their cognitive skills (Shi et al., 2013). Consequently, an early identification of intellectual giftedness appears to be a predictor of not only their psychological well-being (Neihart, 1999; Litster and Roberts, 2011; Kroesbergen et al., 2016), but also their professional success as adults (Rinn and Bishop, 2015).

Intellectual giftedness is usually defined in terms of a high Intellectual Quotient (IQ) resulting from a standardized and validated intelligence test (Winner, 2000; see Lovett and Lewandowski, 2006). The Wechsler Intelligence Scale for Children (WISC-IV; Wechsler, 2005; WISC-V; Wechsler, 2016) is one of the most common tools used by both researchers and clinicians for the evaluation of intellectual capacity (Evers et al., 2012). It is currently used to identify intellectually gifted children in school (McClain and Pfeiffer, 2012). It allows a person's global intellectual potential to be estimated on the basis of a Full Scale Intellectual Quotient (FSIQ). The most commonly considered threshold value of the FSIQ is a score greater than or equal to 130, i.e., 2 standard deviations above the average (Carman, 2013). However, this cutoff is an ideal target value. Very high cutoffs also require a very high level of reliability in the measurement, in order to ensure satisfactory identification performance. Indeed, the measurement error has an influence on the identification of IGC (McIntosh et al., 2005). It perturbs the accuracy of decisions related to their identification (McBee et al., 2013). According to McIntosh et al. (2005), a threshold FSIQ value of 125, i.e., the 95th percentile, appears to be a reasonable choice for the identification of IGC. In the current study, we retained this cutoff of 125 in Wechsler's scale to identify IGC.

The recent versions of Wechsler scales are explicitly developed on the basis of the theoretical model of Carroll-Horn-Cattel (CHC; Keith et al., 2006; Lecerf et al., 2010a; Golay et al., 2013; Weiss et al., 2013). The literature is consensual with regard to the number of global cognitive abilities evaluated by the WISC-IV (Reverte et al., 2015) or WISC-V (Reynolds and Keith, 2017). Subtests are posited to estimate global cognitive aptitudes: *Similarities, Vocabulary*, and *Comprehension* are considered to evaluate the Comprehension-Knowledge (Gc) ability; *Pictures Concept, Figure Weights* (only WISC-V) and *Reasoning Matrix* are considered to estimate Fluid reasoning (Gf) ability; *Block Design* and *Visual Puzzle* (only WISC-V) are posited to evaluate mainly Visual processing (Gv) ability; *Digit Span, Picture Span* (only WISC-V) and *Letter-Number Sequencing* are considered to evaluate Short-memory ability (Gsm), and *Coding* and *Symbol* target processing Speed (Gs) ability.

In practice, IGC have higher performance for Gc, Gf and Gv than for Gsm and Gs (Volker et al., 2006; Rowe et al., 2010). It thus appears that IGC performed more efficiently in terms of high-level abilities than in terms of low-level abilities. The consequence of this is the presence of strong scatter scores in Wechsler scale with IGC. According to Flanagan and Kaufman (2009), an important and uncommon variability can affect the interpretation of the FSIQ. Indeed, the aim of the FSIQ is to summarize all cognitive aptitudes assessed by the scale. The FSIQ score is designed to estimate Spearman's *g* factor 1904. If the tests are too heterogeneous, the common variance between tests decreases and the mean value of the performance does not provide a satisfactory representation of the overall cognitive aptitude. This abnormal variability between scores in Wechsler scale could have an impact on the estimation of a child's overall intellectual functioning (Lecerf et al., 2015). To mitigate this problem, Weiss et al. (2008) proposed a new indicator: the Global Aptitude Index (GAI). This is estimated from subtests used mainly to evaluate Gc, Gf, and Gv abilities. These are the most highly *g*-saturated core subtests (Keith et al., 2006; Lecerf et al., 2010b). The GAI would thus provide a better estimation of overall cognitive functioning when low *g*-loaded cognitive aptitudes are lower than the high *g*-loaded cognitive aptitudes (Watkins et al., 2002; Sattler and Ryan, 2009; Lecerf et al., 2016). Considering the discrepancies in performance for the low- and high-level cognitive abilities in IGC, the use of GAI appears to be more judicious than the FSIQ in the context of the identification of IGC (Newman et al., 2008). The GAI also has a satisfactory reliability with regard to both short- and long-term stability (Kieng et al., 2013; Watkins and Smith, 2013). It is often used to include the gifted children in special education such as enrichment or accelerated programs (Saklofske et al., 2005; Pierson et al., 2012). The GAI is often considered as an abbreviated form of the Wechsler scale for the identification of IGC. However, the short form of a test should reduce the examination time by at least 50% (Levy, 1968). The GAI reduces the administrative time by approximately 23% (Ryan et al., 2007). As a consequence, the identification of IGC often remains time-consuming, even with the GAI.

In order to reduce the examination time of a cognitive ability test, the most commonly used solution described in the literature is to reduce the number of subtests retained to estimate the global cognitive functioning (Silverstein, 1990). This time should not be gained at the cost of predictive accuracy (Doppelt, 1956). The use of short forms comprising more than 4 subtests only weakly increases the reliability of the measurement with respect to the cost in terms of examination time (Karnes and Brown, 1981).

The usefulness of brief intellectual assessment is controversial, because it prevents to analyse the cognitive profile which can be essential in the learning disabilities context (Fiorello et al., 2001; Hale et al., 2007). Nevertheless, the brief intellectual assessment may be useful to estimate the intellectual potential. Indeed, several short forms of the WISC-IV have been validated in various languages (e.g., Crawford et al., 2010; in English; Dasi et al., 2014, in Spanish). These short forms have been used to obtain a rapid and reliable evaluation of intellectual ability in children with an intellectual disability (McKenzie et al., 2013; Murray et al., 2016), children having a high-functioning autism spectrum (Thomeer et al., 2012), children affected by epilepsy (Hrabok et al., 2012) or children affected by traumatic brain injury (Donders et al., 2013). Recent studies have used shortened versions of the Wechsler scale with 2 or 4 subtests, for the detection of IGC (Shaw et al., 2006; Alloway and Elsworth, 2012; van Viersen et al., 2014, 2015). Most of these short forms have been constructed on the basis of subtests evaluating high-level cognitive abilities such as verbal comprehension and perceptive reasoning. Although there are short forms of the previous versions of the Wechsler Intelligence Scale for Children (WISC-R and WISC-III) as well as other sets of cognitive ability evaluations for the identification of IGC (Killan and Hughes, 1978; Dirks et al., 1980; Karnes and Brown, 1981; Ortiz and Gonzalez, 1989; Mark et al., 1998; for a review Simpson et al., 2002; Reiter, 2004; Pierson et al., 2012), to our knowledge, no short form of the WISC-IV or WISC-V has been tested with respect to its psychometric qualities in this atypically developing population. With respect to the use of shortened tests of cognitive ability as a decisional aid for the identification of IGC, Prewett (1995) suggests that shortened tests should generate scores that are comparable with those obtained with a battery of global assessment tests. It is thus necessary to compare the mean values of the short scale with that of the full scale. If a discrepancy between the IQ short form (IQ_SF_) and the FSIQ is within 2 standard errors of measurement (SEM), the IQ_SF_ is considered as stable (Kieng et al., 2013; Meyers et al., 2013).

In the literature, some authors use 2 or 4 subtests to estimate IQ_SF_ score from Wechsler scale to identify IGC (Shaw et al., 2006; Alloway and Elsworth, 2012; van Viersen et al., 2014, 2015). To our knowledge, the psychometric qualities of these short forms have never been tested for the identification of IGC. In the present study, we make nine IQ_SF_ from all possible combinations of 2 or 4 subtests in Verbal Comprehension Index (VCI) and Performance Reasoning Index (PRI). We compare reliability, validity, and screening qualities with each other. We excluded the subtests involving the working memory, because these subtests are also known to be affected negatively by specific learning disabilities (Maehler and Schuchardt, 2009; Cornoldi et al., 2014; Toffalini et al., 2017a). While IGC are known to elicit high performances in working memory tasks (Calero et al., 2007; Hoard et al., 2008; Ruthsatz and Urbach, 2012), these subtests can underestimate the overall cognitive performance in the context of specific learning disability. All short forms based on subtests from VCI and PRI make it possible to obtain an estimation of the FSIQ and GAI scores in less than 30 min. The aim of our study is thus to determine the best short scales, in terms of their psychometric qualities, for the identification of IGC.

## Methods

### Participants

The data was collected from the WISC-IV produced by 117 French IGC (mean age: 10.39, SD: 1.03, 74% boys) and 52 French intellectually typical children (mean age: 10.57, SD: 2.66, 79% boys). Clinician psychologists gave us the data completely anonymous and in the respect of French deontological code. To preserve the anonymity of the children, we chose not to contact the parents and their children. The groups did not significantly differ in terms of age, *t*_(57.902)_ = −0.485, *p* = 0.629, *d* = −0.109, or gender, $\chi_{\left(1\right)}^{2}$ = 0.550, *p* = 0.458. The children were contacted through teachers, school psychologists or licensed psychologists. In order to allow for measurement errors, the inclusion criterion for intellectually gifted group was set to FSIQ or GAI score greater than or equal to 125 in accordance with the current recommendation (McIntosh et al., 2005; Assouline et al., 2010; Brasseur and Grégoire, 2010).

### Procedure

All of the statistical analyses were made using version 3.4.2 of R software (R Core Team, 2017). We estimated the interclass correlation of each IQ_SF_ and the full-scale using the *irr* library (Gamer et al., 2012) and the hierarchical omega (ω_*H*_) of each IQ_SF_ using the *MBESS* library (Kelley, 2018). We also computed the 95% confidence intervals for the correlation comparisons (Zou, 2007) using the *cocor* library (Diedenhofen and Musch, 2015).

The data relevant to the preparation of short forms was extracted from fully administered WISC-IV protocols. The FSIQ scores were computed from 10 core subtests from the WISC-IV. The GAI scores were obtained from the VCI and PRI (Lecerf et al., 2010a).

The current study is composed of three steps in our statistical analyses. Firstly, the scores from different IQ_SF_ were computed using the linear equation method (Tellegen and Briggs, 1967). They were divided by the sum of the standard scores given by the VCI (Vocabulary [Vo], Similarities [Si] and Comprehension [Co]) and PRI (Block Design [Bd], Picture Concepts [Pc], and Matrix Reasoning [Mr]). Secondly, we realized a simulation of 1,000,000 data with the Choleski decomposition from correlation matrix of WISC-IV (Table 4.1; Wechsler, 2005) using the *mvtnorm* library (Genz et al., 2018) (see Giofrè et al., 2017 for a similar approach). We realized a comparison between psychometrics parameters (i.e., reliability and validity) estimated from empirical and simulated datasets. Then, we selected the three best IQ_SF_ based on these psychometrics parameters estimated from simulated dataset. For each selected short form WISC-IV, we computed the indicators of a receiver operating characteristic (ROC) such as the sensitivity, the sensibility, the false positive rate (FPR), the false negative rate (FNR), and the Area Under Curve (AUC) from our empirical sample using the *pROC* library (Robin et al., 2011). The AUC is used as a general measure to estimate the performance of the IGC classification (Fawcett, 2006). Thirdly, we realized another simulated dataset from correlation matrix of WISC-V (Table 4.1; Wechsler, 2016). From this simulated dataset, we created the different short forms of WISC-V. We then highlighted the three best short forms of WISC-V based on the estimated psychometrics parameters from simulated dataset.

The data and R script used for the simulations and the statistical analyses are available on the Open Science Framework (OSF) at http://osf.io/dax8p.

### Statistical analyses

Nine IQ_SF_ derived from 2-subtest forms, and 9 IQ_SF_ derived from 4-subtest forms were computed from 3 PRI and 3 VCI subtests. For each IQ_SF_, the threshold of intellectual giftedness identification was superior to 125, i.e., the 95th percentile.

It is essential to evaluate reliability and validity when selecting the best short form (Cyr and Brooker, 1984). For each form that we prepared, reliability and validity indices were thus computed.

#### Index of reliability

##### Alpha

The reliability of each form was determined by a composite reliability coefficient (r_cc_) according to Equation (1) from Tellegen and Briggs (1967), based on a table of internal consistency and inter-correlations of the applied subtests derived from the WISC-IV manual (Table 4.1 and Table 5.1 from Wechsler, 2005) and the WISC-V manual (Table 4.1 and Table 5.1 from Wechsler, 2016). This index allows the standard error of each short form measurement to be determined. The composite reliability coefficient is frequently used to estimate the reliability of the abriged scale (Ryan and Ward, 1999; Girard et al., 2010, 2015; Donders et al., 2013; Denney et al., 2015).

$$\begin{array}{l} r_{cc}=\frac{\sum r_{jj}+2\times \sum r_{jk}}{n+2\times \sum r_{jk}} \end{array}$$

where *r*_*jj*_ is the reliability coefficient of the jth subtest in IQ_SF_, *n* is the number of subtests in IQ_SF_, *r*_*jk*_ is the correlation coefficient between the jth subtest and the kth subtest used for IQ_SF_.

##### Hierarchical omega

In contrast to the coefficient alpha reliability, the coefficient omega (ω) takes into account the unequal factor loadings (Watkins, 2017). In particular, the hierarchical omega (ω_*H*_) has also the advantage to be unaffected by the fitting factorial analysis model (Kelley and Pornprasertmanit, 2016). So, ω_*H*_ can be a better index of the reliability of a composite score from Wechsler scale than alpha coefficient (Gignac and Watkins, 2013). This index estimates the variation portion which is involved by the general factor. High value of ω_*H*_ indicates that a general factor explains a large part of variation in the composite score. ω_*H*_ coefficient is considered as reliable if it exceeds 0.50 at minimum, but ω_*H*_ superior or equal to 0.75 is considered better (Reise et al., 2013).

##### Interclass correlation coefficient

In addition, we used the Interclass Correlation Coefficient (ICC; model A.1 in McGraw and Wong, 1996) to examine the reliability of both the IQ_SF_ and the full-scale scores.

#### Index of validity

Three validity indices were estimated: the convergent validity determined by the corrected correlation between the short form and the full form of the scale (*r*′), the degree of discrepancy computed as the difference between the IQ_SF_ score and the full form score (FSIQ or GAI), and the accuracy of the estimation of the full form scores (C_acc_).

##### Convergent validity

The convergent validity of each form was determined by computing its correlation (Pearson's *r*) with the FSIQ and GAI scores. These correlations were then corrected (*r*′; Equation 2) by taking the redundancy of the variance error into account, using the modified version (Girard and Christensen, 2008) of the Levy formula (Levy, 1967). The forms were indeed prepared from 2 or 4 tests taken from the entirely administered scale. The correlation between the forms and the full forms (FSIQ or GAI) is artificially increased by the measurement error shared between the two forms:

$$\begin{array}{l} r^{\prime }=r_{sf}-\left(1-r_{cc}\right)\times \frac{\sqrt{p+2\times r_{jk}}}{\sqrt{10+2\times r_{lm}}}\times \frac{SD_{sf}}{15} \end{array}$$

where *r*′ is the corrected correlation coefficient; *r*_sf_ is the uncorrected correlation coefficient between the score of the short form (QI_SF_) and the full scale (FSIQ or GAI), *r*_*cc*_ is the composite reliability coefficient of QI_SF_, *r*_*jk*_ is the correlation coefficient between the jth subtest and the kth subtest used for IQ_SF_, *r*_*lm*_ is the correlation coefficient between subtest l and subtest m, used for the FSIQ or the GAI, *p* is the number of subtests used for the IQ_SF_, SD_SF_ is the standard deviation of the QI_SF_.

##### Degree of discrepancy

The paired Student *t*-tests were computed in order to determine whether the average of the QI_SF_ scores was significantly different at the average of the FSIQ or GAI scores. This index allows us to define whether the measurement provided by the scale is significantly different at that of the full scale. The extent of this effect (Cohen's *d* for correlated samples comparison; Lakens, 2013) was estimated, in order to determine the magnitude of the difference in mean computed value.

##### Accuracy

An indicator of the accuracy (C_acc_) of the estimation of FSIQ or GAI from the QI_SF_ score was prepared, in order to identify the percentage of individuals in our sample having a QI_SF_ score greater than or equal to the threshold value of 125. The indicator C_acc_ also takes into account the measurement stability between the QI_SF_ score and the FSIQ and GAI scores. Usually, this stability is determined by the difference between two scores in the range between −2 and +2 standard errors of measurement (Meyers et al., 2013). C_acc_ is a coefficient lying in the range between 0 and 1. It can be interpreted as the accuracy of IGC identification. The closer C_acc_ is to 1, the more accurate the QI_SF_ score-based identification.

In order to simplify the interpretation of multiple psychometric reliability and validity scores, we construct a composite indicator (R_c_) based on the approach proposed by Cyr and Brooker (1984), and adapted by Girard and Christensen (2008). R_c_ corresponds to the unweighted average of 4 computed reliability and validity indicators, i.e., the composite reliability coefficient (*r*_*cc*_), the hierarchical omega (ω_*H*_), the interclass correlation coefficient (ICC), the corrected correlation (*r*′) between the IQ_SF_ score and the FSIQ and GAI scores, the identification accuracy (C_acc_). We thus reproduced and adapted the psychometric agreement indicator of Girard and Christensen (2008), with our forms and the full form of the Wechsler scale. This makes it possible to interpret the strength of agreement, which ranges from 0 (absence) to 1 (perfect), between the IQ_SF_ score and the FSIQ and GAI scores (Girard et al., 2015).

## Results

### Description of the sample

All of the participants obtained a GAI score equal to, or greater than 125. The descriptive data from our sample, as well as the mean and standard deviation of the FSIQ, the GAI, and the substests from WISC-IV, are shown in Table 1.

**Table 1:** Descriptive statistics of the sample.

	**Intellectually gifted children (*****n*** = **117)**			**Typical children (*****n*** = **52)**			***d_*S*_*^a^**
**Variables**	**M (SD)**	**Skew**.	**Kurt**.	**M (SD)**	**Skew**.	**Kurt**.	
Age (in months)	124.65 (12.34)	0.31	−0.99	126.87 (31.88)	0.43	−0.89	−0.109
Gender (%)							
Male	73.50	–	–	78.85	–	–	
Female	26.50	–	–	21.15	–	–	
WISC-IV							
Block design	13.46 (2.62)	0.43	−0.27	9.85 (2.67)	0.46	0.46	1.372^**^
Similarities	17.17 (1.99)	−0.65	−0.96	12.48 (2.32)	0.31	−0.19	2.239^**^
Digit span	12.97 (2.94)	0.19	−0.19	10.08 (2.68)	0.11	−0.44	1.013^**^
Picture concepts	14.30 (2.21)	0.82	0.20	9.44 (2.34)	0.00	−1.06	2.159^**^
Vocabulary	16.44 (2.02)	−0.35	−0.83	12.25 (2.22)	0.11	−0.71	2.014^**^
LNS	13.13 (2.28)	0.06	0.18	10.15 (2.73)	−0.47	1.01	1.228^**^
Matrix reasoning	13.72 (2.38)	−0.25	0.20	10.75 (1.79)	−0.17	−0.77	1.338^**^
Comprehension	15.63 (2.44)	−0.29	−0.71	11.58 (2.84)	−0.63	2.06	1.579^**^
Coding	11.26 (2.96)	−0.06	−0.05	9.06 (3.08)	0.45	0.15	0.734^**^
Symbol	11.72 (3.00)	0.07	0.14	9.69 (3.46)	−0.52	0.00	0.643^**^
FSIQ	134.92 (9.43)	0.03	−0.82	104.37 (8.35)	−0.33	−0.68	3.353^**^
GAI	137.09 (7.66)	0.20	−0.29	107.58 (5.84)	−0.88	−0.15	4.127^**^

*N = 169; LNS, Letter-Number Sequencing; VCI, Verbal Comprehension Index; PRI, Performance Reasoning Index; WMI, Working Memory Index; PSI, Processing Speed Index; FSIQ, Full Scale Intellectual Quotient; GAI, General Aptitude Index*;

** *p < 0.01 with Bonferroni correction*.

a *Cohen's d for two independent samples comparison (Equation 1 from Lakens, 2013)*.

The deviations and equations for each IQ_SF_ are presented in Table 2. All of the scores from each 2-subtest and 4-subtest IQ_SF_ have skewness and kurtosis coefficient lying between −1 and 1. All of the 2- and 4-subtests forms have an average value greater than 125 in intellectually gifted children and an average value of 107 in typical children. The raw data is available in the Supplementary Material (Data Sheet 1).

**Table 2:** Mean (SD) of WISC-IV Short-Form Scores and formulas.

	**Intellectually gifted children (*****n*** = **117)**			**Typical children (*****n*** = **52)**			***d_*S*_*^a^**	**Statistical formulas^a^**
**Short Form**	**M (SD)**	**Skew**	**Kurt**	**M (SD)**	**Skew**	**Kurt**		
2 Subtests								
SiMr	132.77 (9.48)	−0.04	−0.56	109.72 (8.16)	−0.32	0.15	2.533^**^	3.01 × (Si+Mr) + 39.81
SiPc	135.03 (8.83)	−0.34	−0.06	105.87 (7.95)	−0.01	−0.77	3.402^**^	3.05 × (Si+Pc) + 38.92
SiBd	132.12 (9.45)	0.16	−0.02	107.03 (10.16)	−0.24	−0.21	2.594^**^	3.02 × (Si+Bd) + 39.59
VoMr	130.47 (9.56)	−0.28	0.06	109.00 (7.39)	−0.58	0.30	2.400^**^	3.00 × (Vo+Mr) + 40.02
VoPc	132.69 (8.89)	−0.23	−0.40	105.15 (9.05)	−0.15	0.16	3.081^**^	3.04 × (Vo+Pc) + 39.14
VoBd	130.48 (9.93)	−0.04	0.31	106.45 (9.27)	0.13	−0.36	2.469^**^	3.08 × (Vo+Bd) + 38.45
CoMr	129.22 (10.27)	0.20	−0.22	107.27 (8.81)	−0.62	0.53	2.229^**^	3.13 × (Co+Mr) + 37.50
CoPc	130.92 (10.93)	−0.18	−0.04	103.17 (8.71)	−0.54	−0.27	2.693^**^	3.11 × (Co+Pc) + 37.74
CoBd	129.23 (11.97)	0.29	−0.47	104.57 (9.01)	−0.11	−0.25	2.212^**^	3.21 × (Co+Bd) + 35.72
4 Subtests								
SiVoMrPc	136.05 (7.83)	−0.16	−0.10	108.21 (7.21)	−0.23	−0.82	3.643^**^	1.67 × (Si+Vo+Mr+Pc) + 33.33
SiVoMrBd	134.54 (8.08)	0.09	−0.35	108.85 (7.87)	−0.46	−0.53	3.206^**^	1.66 × (Si+Vo+Mr+Bd) + 33.55
SiVoPcBd	136.40 (7.40)	0.17	−0.06	106.84 (7.74)	−0.34	−0.88	3.939^**^	1.70 × (Si+Vo+Pc+Bd) + 31.88
SiCoMrPc	135.92 (8.42)	0.12	−0.10	107.33 (6.36)	−0.52	−0.50	3.641^**^	1.73 × (Si+Co+Mr+Pc) + 30.99
SiCoMrBd	134.56 (8.67)	0.22	−0.45	108.05 (7.15)	−0.48	−0.69	3.220^**^	1.73 × (Si+Co+Mr+Bd) + 30.83
SiCoPcBd	136.31 (8.55)	−0.02	−0.29	105.91 (6.22)	−0.75	−0.08	3.841^**^	1.77 × (SI+Co+Pc+Bd) + 29.38
VoCoMrPc	134.50 (8.73)	0.09	−0.32	106.90 (6.57)	−0.62	0.23	3.394^**^	1.72 × (Vo+Co+Mr+Pc) + 31.32
VoCoMrBd	133.38 (9.10)	0.40	−0.42	107.67 (6.44)	−0.58	−0.66	3.068^**^	1.73 × (Vo+Co+Mr+Bd) + 30.66
VoCoPcBd	135.11 (9.01)	0.12	−0.39	105.51 (6.26)	−0.72	0.72	3.581^**^	1.77 × (Vo+Co+Pc+Bd) + 29.20

*n = 169; Si, Similitaries; Vo, Vocabulary; Co, Comprehension; Mr, Matrix Reasoning; Pc, Pictures Concept; Bd, Block Design*;

** *p < 0.01 with Bonferroni correction*.

a *Cohen's d for two independent sample (Equation 1 from Lakens, 2013)*.

b *Equation (4) from Tellegen and Briggs (1967)*.

### Short form WISC-IV estimation with simulated dataset

We realized a comparison between the reliability and validity indicators from real and simulated data in WISC-IV. The composite indicator Rc calculated with our sample and the simulated dataset are highly correlated [*r*_(36)_ = 0.988]. In addition, there are few differences between each indicator of reliability and validity estimated with our sample and these with the simulated dataset have few differences (Table 3). The near perfect duplication of psychometric quality indicators shows that their estimation with simulated data is very close to those derived from real data. We thus selected the short forms from the simulated dataset.

**Table 3:** Comparison the principal indexes of reliability and validity estimated with our sample and the simulated dataset.

	**FSIQ**			**GAI**			**Rc**
	***r*′**	**C_acc_**	**ICC**	***r*′**	**C_acc_**	**ICC**	
Difference	−0.013	−0.017	0.003	−0.010	0.015	0.014	−0.001
*r*	0.906^**^	0.831^**^	0.976^**^	0.981^**^	0.909^**^	0.974^**^	0.988^**^

*N = 36*.

** *;p < 0.01*.

The 2-subtest and 4-subtest forms are ranked by decreasing value of the composite score R_c_, determined to use the reliability index and each validity index (see Table 4). All of the IQ_SF_ scores produced by the 2 subtests are significantly correlated with the FSIQ, $r_{\left(1\text{e}6\right)}^{\prime }\in$ [0.680; 0.786]; *p* < 0.01. All of the IQ_SF_ scores computed from 4 subtests are also correlated with the FSIQ, *r*′_(1e6)_ ∈ [0.823; 0.851]; *p* < 0.01. However, the forms with 4 subtests are significantly more strongly correlated with the FSIQ, respectively *r*_(9e6)_ = 0.791; *r*_(9e6)_ = 0.885; 95% CI [−0.094, −0.094], and the GAI, respectively *r*_(9e6)_ = 0.856; *r*_(9e6)_ = 0.957; 95% CI [−0.102, −0.102], than with the forms with 2 subtests

**Table 4:** Reliability and Validity of WISC-IV Short Forms from simulated data.

**Short form**	**r_cc_**	**ω_*H*_**	**FSIQ**			**GAI**			**R_c_**
			***r′***	**ICC**	**C_acc_**	***r′***	**ICC**	**C_acc_**	
2 Subtests									
SiMr	**0.877**	**0.550**	**0.786**	**0.820**	0.797	**0.833**	**0.882**	**0.909**	**0.807**
VoMr	**0.871**	**0.561**	**0.783**	**0.818**	0.804	**0.826**	**0.877**	0.907	**0.806**
VoBd	0.845	0.484	**0.771**	**0.813**	**0.828**	**0.801**	0.862	0.907	**0.789**
SiBd	**0.858**	**0.540**	0.759	0.797	0.791	0.791	0.848	0.872	0.782
SiPc	0.825	0.507	0.741	0.788	0.813	0.795	**0.864**	**0.919**	0.781
VoPc	0.819	0.518	0.737	0.786	**0.818**	0.788	0.860	**0.918**	0.781
CoMr	0.836	0.437	0.741	0.784	0.795	0.790	0.853	0.897	0.767
CoBd	0.806	0.347	0.729	0.779	**0.820**	0.765	0.837	0.898	0.748
CoPc	0.787	0.449	0.680	0.736	0.786	0.737	0.819	0.882	0.734
4 Subtests									
SiVoMrBd	**0.917**	**0.733**	**0.851**	**0.891**	0.856	**0.892**	0.952	0.987	**0.885**
SiVoPcBd	0.898	**0.723**	0.840	0.889	0.874	0.887	0.959	0.996	**0.883**
SiVoMrPc	**0.908**	**0.733**	0.839	0.884	0.851	**0.893**	**0.959**	0.994	**0.883**
VoCoMrBd	0.900	0.684	**0.845**	**0.893**	**0.879**	0.889	0.958	0.995	0.881
SiCoMrBd	**0.903**	0.692	**0.844**	**0.890**	0.871	**0.890**	0.957	0.994	0.880
VoCoPcBd	0.880	0.672	0.830	0.886	**0.888**	0.879	**0.961**	**0.998**	0.874
SiCoMrPc	0.894	0.694	0.827	0.878	0.855	0.885	0.959	**0.996**	0.874
SiCoPcBd	0.883	0.677	0.829	0.883	**0.881**	0.880	**0.959**	**0.997**	0.873
VoCoMrPc	0.893	0.700	0.823	0.874	0.852	0.879	0.954	0.993	0.871

*N = 1,000,000. Si, Similitaries; Vo, Vocabulary; Co, Comprehension; Mr, Matrix Reasoning; Pc, Pictures Concept; Bd, Block Design. Boldfaced values highlight the top three short-forms according to each psychometric measure. All corrected correlation coefficient (r′) are significant at p < 0.01*.

The short form with the *Similarities* + *Matrix Reasoning* (SiMr) subtests has the highest agreement score of all 2 subtest forms, R_c_ = 0.807. It is most strongly correlated with the FSIQ, *r*′_(1e6)_ = 0.786; *p* < 0.01, and GAI score, *r*′_(1e6)_ = 0.833; *p* < 0.01. In 80% of cases, the IQ_SF_ score for the SiMr form correctly identifies the IGC in our sample, and their IQ_SF_ lie within 2 standard errors of measurement of the FSIQ. In 91% of cases, the IQ_SF_ score for the SiMr form correctly identifies the IGC in our sample, and their IQ_SF_ lie within 2 standard errors of measurement of the GAI.

Among the 4-subtest forms, the IQ_SF_ scores of *Similarities* + *Vocabulary* + *Matrix Reasoning* + *Block Design* [SiVoMrBd] form has a higher accession score, R_c_ = 0.885. It is strongly correlated with the FSIQ, *r*′_(1e6)_ = 0.851; *p* < 0.01, and GAI score, *r*′_(1e6)_ = 0.892; *p* < 0.01. In 86% of cases, the IQ_SF_ score for the SiVoMrBd form correctly identifies the IGC in our sample, and their IQ_SF_ lie within 2 standard errors of measurement of the FSIQ. In 99% of cases, the IQ_SF_ score for the SiVoMrBd form correctly identifies the IGC in our sample, and their IQ_SF_ lie within 2 standard errors of measurement of the GAI.

### Identification efficiency of short-form WISC-IV from empirical data

After selecting the three best short forms with 2- and 4-subtests from simulated dataset, we realized the AUC, sensitivity and sensibility of the three best IQ_SF_ from empirical dataset. In Table 5, the short forms with 2 subtests and 4 subtests are ranked in descending order of the AUC indicating the predictive performance in identifying the IGC. All short form scales have a high AUCs suggesting a highly predictive performance with 2- and 4-subtests models. The difference among all models is low (Δ*AUCs* < 0.05).

**Table 5:** Performance analysis of three best 2- and 4-subtests WISC-IV short-forms.

**ShortForm**	**Skew**	**Kurt**	**FSIQ**		**GAI**		**IQ**_**SF**_ ≥ **125**				
			***t***	***d_rm_*^a^**	***t***	***d_rm_*^a^**	**AUC**	**Sensitivity**	**Specificity**	**FPR**	**FNR**
2 Subtests											
SiMr	−0.268	−0.585	**0,215**	**0.010**	−3.903^**^	**−0.156**	**0.970**	**0.744**	**0.981**	**0.019**	**0.256**
VoMr	−0.230	−0.609	−2,253^*^	−0.103	−7.102^**^	−0.278	0.962	0.718	0.981	0.019	0.282
VoBd	−0.289	−0.524	−3,216^**^	−0.151	−8.305^**^	−0.325	0.958	0.675	0.962	0.038	0.325
4 Subtests											
SiVoPcBd	−0.335	−0.685	3.053^*^	0.109	−2.367	−0.045	**0.999**	**0.957**	**1.000**	**0.000**	**0.043**
SiVoMrPc	−0.180	−0.684	3.240^**^	0.120	**−1.486**	**−0.035**	0.996	0.906	1.000	0.000	0.094
SiVoMrBd	−0.466	−0.540	**1.929**	**0.068**	−3.726^**^	−0.090	0.994	0.880	1.000	0.000	0.120

*N = 169. Si, Similitaries; Vo, Vocabulary; Co, Comprehension; Mr, Matrix Reasoning; Bd, Block Design; AUC, Area Under Curve; FPR, False Positive Rate; FNR; False Negative Rate; Boldfaced values highlight the top of short-forms according to each psychometric measure*.

* *p < 0.05*;

** *p < 0.01*.

a *Cohen's d for correlated samples comparison (Equation 9 from Lakens, 2013)*.

Among the forms with 2 subtests, the form with the best performance is still *Similitaries* + *Matrix Reasoning* [SiMr]. They allow more than 74% of IGC in our sample to be correctly identified. The SiMr form has the probability of around 1.1% typical children being incorrectly identified as gifted. It has only significant difference with GAI, *t*_(168)_ = −3.903, *p* < 0.01, *d*_*rm*_ = −0.156, but it has the smallest effect size among the three best 2-subtests short forms.

Among the forms comprising 4 subtests, the IQ_SF_ with the best performance consisted of the *Similarities* + *Vocabulary* + *Picture Concept* + *Block Design* [SiVoPcBd] subtests. It correctly identifies more than 96% of IGC in our sample. No typical children were identified as being gifted, but 4.3% IGC from our sample have not been correctly identified. It has only a significant difference with FSIQ, *t*_(168)_ = −3.053, *p* < 0.05, *d*_*rm*_ = −0.109.

### Short-form WISC-V estimation from simulated dataset

The 2-subtest and 4-subtest forms are ranked by decreasing value of the composite score R_c_ (see Table 6). All of the IQ_SF_ scores produced by the 2 subtests are significantly correlated with the FSIQ, *r*′_(1e6)_ ∈ [0.741; 0.833]; *p* < 0.01. All of the IQ_SF_ scores computed from 4 subtests are also correlated with the FSIQ, *r*′_(1e6)_ ∈ [0.921; 0.940]; *p* < 0.01.

**Table 6:** Reliability and Validity of WISC-V Short Forms from simulated data.

**Short Form**	**Statistical formulas^a^**	**r_cc_**	**ω_*H*_**	**FSIQ**			**GAI**			
				***r′***	**ICC**	**C_acc_**	***r′***	**ICC**	**C_acc_**	**R_c_**
2 Subtests										
SiMr	2.91 + (Si+Mr) + 41.88	0.885	**0.648**	**0.833**	**0.874**	**0.866**	**0.855**	**0.907**	**0.922**	**0.849**
SiBd	2.95 + (Si+Bd) + 41.07	0.875	**0.611**	**0.826**	**0.870**	**0.870**	0.840	0.896	**0.913**	**0.838**
SiFw	2.93 + (Si+Fw) + 41.48	**0.911**	**0.630**	**0.829**	0.861	0.804	**0.861**	**0.901**	0.880	**0.835**
VoMr	2.99 + (Vo+Mr) + 40.24	0.882	0.571	0.826	**0.866**	0.856	**0.849**	**0.901**	**0.914**	0.833
VoBd	3.03 + (Vo+Bd) + 39.37	0.871	0.529	0.819	0.863	**0.863**	0.834	0.89	0.904	0.822
VoFw	2.97 + (Vo+Fw) + 40.66	**0.912**	0.591	0.810	0.841	0.766	0.843	0.882	0.836	0.810
CoMr	2.99 + (Co+Mr) + 40.24	0.868	0.571	0.741	0.786	0.744	0.735	0.793	0.753	0.749
CoBd	3.10 + (Co+Bd) + 37.98	0.850	0.461	0.750	0.799	0.785	0.734	0.798	0.784	0.745
CoFw	3.05 + (Co+Fw) + 38.92	**0.892**	0.507	0.747	0.784	0.707	0.751	0.798	0.724	0.739
4 Subtests										
SiVoMrBd	1.61 + (Si+Vo+Mr+Bd) + 35.52	0.928	**0.773**	**0.898**	**0.943**	**0.948**	**0.917**	**0.976**	**0.998**	**0.923**
SiVoFwBd	1.63 + (Si+Vo+Fw+Bd) + 34.97	**0.936**	**0.767**	**0.900**	**0.941**	**0.934**	**0.925**	**0.978**	**0.999**	**0.922**
SiVoMrFw	1.60 + (Si+Vo+Mr+Fw) + 35.98	**0.940**	**0.782**	**0.896**	**0.935**	**0.911**	**0.925**	**0.975**	**0.997**	**0.920**
SiCoFwBd	1.67 + (Si+Co+Fw+Bd) + 33.18	0.927	0.741	0.877	0.922	0.906	0.887	0.944	0.959	0.895
SiCoMrBd	1.64 + (Si+Co+Mr+Bd) + 34.35	0.921	0.762	0.867	0.917	0.902	0.871	0.935	0.944	0.890
SiCoMrFw	1.63 + (Si+Co+Mr+Fw) + 34.70	**0.933**	0.767	0.867	0.909	0.869	0.881	0.936	0.934	0.887
VoCoFwBd	1.68 + (Vo+Co+Fw+Bd) + 32.66	0.927	0.723	0.867	0.911	0.884	0.877	0.934	0.937	0.883
VoCoMrBd	1.66 + (Vo+Co+Mr+Bd) + 33.55	0.921	0.740	0.860	0.910	0.890	0.865	0.929	0.930	0.881
VoCoMrFw	1.64 + (Vo+Co+Mr+Fw) + 34.20	0.933	0.753	0.857	0.899	0.846	0.871	0.926	0.910	0.874

*N = 1,000,000. Si, Similitaries; Vo, Vocabulary; Co, Comprehension; Mr, Matrix Reasoning; Fw, Figure Weights; Bd, Block Design. Boldfaced values highlight the top three short-forms according to each psychometric measure. All corrected correlation coefficient (r′) are significant at p < 0.01*.

a *Equation (4) from Tellegen and Briggs (1967)*.

Like at the WISC-IV, the short form with the *Similarities* + *Matrix Reasoning* [SiMr] subtests has the highest agreement score of all 2 subtest forms, R_c_ = 0.849. It is strongly correlated with the FSIQ, *r*′_(1e6)_ = 0.833; *p* < 0.01, and GAI score, *r*′_(1e6)_ = 0.855; *p* < 0.01. In 87% of cases, the IQ_SF_ score for the SiMr form correctly identifies the IGC in our sample, and their IQ_SF_ lie within 2 standard errors of measurement of the FSIQ. In 92% of cases, the IQ_SF_ score for the SiMr form correctly identifies the IGC in our sample, and their IQ_SF_ lie within 2 standard errors of measurement of the GAI.

Like at the WISC-IV, the *Similarities* + *Vocabulary* + *Matrix Reasoning* + *Block Design* [SiVoMrBd] form has a high agreement score, R_c_ = 0.923. It is strongly correlated with the FSIQ, *r*′_(1e6)_ = 0.898; *p* < 0.01, and GAI score, *r*′_(1e6)_ = 0.917; *p* < 0.01. In 95% of cases, the IQ_SF_ score for the SiVoMrBd form correctly identifies the IGC in our sample, and their IQ_SF_ lie within 2 standard errors of measurement of the FSIQ. In 99.8% of cases, the IQ_SF_ score for the SiVoMrBd form correctly identifies the IGC in our sample, and their IQ_SF_ lie within 2 standard errors of measurement of the GAI.

## Discussion

Our aim was to make a short form of the recent versions of Wechsler scales, allowing an intellectual capacity assessment in less than 30 min. In the literature, short forms with 2 or 4 subtests are often used to estimate intellectual capacity. We thus tested all possible short form combinations, comprising either 2 or 4 subtests used for the evaluation of high-level cognitive abilities.

The results of the present study indicate that the estimation of reliability and validity indicators with simulated data are very close to them estimated with real data. Ours findings also show the short forms of the WISC-IV can have high performance to identify children on having intellectual giftedness. The 4-subtest forms appear to produce better psychometric results than the 2-subtest forms. In addition, the 4-subtest short forms appear to provide a good compromise between test duration and psychometric qualities, which are more satisfactory than those obtained with 2-subtest forms. The ω_*H*_ coefficient also showed that the general factor explained more variance in the 4-subtest than 2-subtest forms. The 4-subtest forms seemed to be a satisfactory trade-off between an accurate estimation of overall cognitive aptitude and administration time (Gignac, 2015).

In spite of a number changes, our results show that the 4-subtest form *Similarities* + *Vocabulary* + *Matrix Reasoning* + *Block Design* [SiVoMrBd], in the WISC-IV and WISC-V, is globally efficient in the identification of IGC. It appears to evaluate the three most discriminating cognitive abilities in the identification of IGC, i.e., Knowledge-Comprehension, Fluid reasoning, and Visual processing (Volker et al., 2006). Moreover, the composite score using the *Similarities* and *Matrix Reasoning* (SiMr) subtests, in the two recent Wechsler scale, appears to provide one of the best 2-subtest forms for the identification of IGC. These two types of short form thus appear to provide acceptable means of identification, for the selection of candidates for complementary evaluations (Prewett, 1995).

In the context of learning disabilities, an abbreviated intellectual assessment using *Similarities, Vocabulary, Matrix Reasoning*, and *Block Design* subtests can allow clinician psychologists to be less biased an overall cognitive aptitude estimation. Indeed, these subtests seem to be less affected by the specific learning disabilities (e.g., Toffalini et al., 2017a,b). The time gain allows us to add another cognitive assessments such as the complex span tasks to assess working memory capacity (e.g., Gonthier et al., 2017).

The Gc, Gf, and Gv abilities evaluated by the subtests of the GAI score appear to provide the best discrimination in the identification of IGC. This is based on the idea that these cognitive abilities appear to be the characteristics of high intellectual potential (Margulies and Floyd, 2009). Thus, psychologists should choose measurements that are well adapted to characteristics that are related to intellectual giftedness (Pierson et al., 2012).

Our results reveal the importance of relying on a theoretical model of cognitive ability, with the aim of identifying IGC as a CHC model. In addition, this theoretical model can be very helpful for the schooling of children in general (Aubry and Bourdin, 2016) and IGC in particular (Warne, 2016).

Any decision in this respect should not be made solely on the basis of the composite score from the short form. It is also important to recognize the reality of measurement errors, which can prevent the correct identification of IGC (Pierson et al., 2012). It is thus recommended to implement a multidimensional evaluation of IGC's characteristics (McClain and Pfeiffer, 2012).

We have shown that some short forms have satisfactory psychometric qualities. However, they have to be accompanied by other assessments such as a teacher rating scale, such as the Gifted Rating Scales–School Form (GRS; Pfeiffer and Jarosewich, 2003) in order to improve the quality of IGC identification (McBee et al., 2013, 2016). Short measurements appear to be reasonable tools for the prediction of global scores of complete batteries of tests, for the identification of children with intellectual giftedness (Newton et al., 2008).

## Limitations of our study

Our simulation of data is very close to our empirical data. However, their relation is not perfect. So, it may have some difference with empirical data for WISC-V short forms. In terms of future perspectives, it would be interesting to implement a detailed analysis of the specificity and sensitivity of each short form of WISC-V identified as being reliable and valid for the identification of IGC.

Among the limitations of our study, the relatively small number of typical children in our sample means that our results must be considered with caution. We tried to estimate the AUC, sensitivity and specificity of all short forms scale.

## Conclusion

The identification of IGC based on the use of standardized tests such as the Wechsler scale is often time-consuming. This drawback can prevent other evaluations from being made, which could be essential for the education of these children with specific needs. The development of a short form of the recent version of Wechsler scale is thus useful for the fast and efficient identification of IGC. This short form would then allow the need for a more in-depth evaluation of various cognitive and socioemotional characteristics to be determined.

Our study evaluated nine 2-subtest forms and nine 4-subtest forms, based on the linear method of Tellegen and Briggs (1967). In order to evaluate these different short forms, we computed with simulated datasets several psychometric indicators that were reorganized into groups with an agreement indicator for FSIQ and GAI scores. We also computed the AUC, sensibility and specificity, on the basis of the 117 IGC and 52 typical children in our sample.

Our results show that the 4-subtest short form at the WISC-IV and WISC-V, *Similarities* + *Vocabulary* + *Matrix Reasoning* + *Block Design* [SiVoMrBd], appears to be one of the most reliable forms for the identification of IGC. Among the 2-subtest forms at the WISC-IV and WISC-V, the *Similarities* + *Matrix Reasoning* [SiMr] version appears to ensure an optimal compromise between reliability and accuracy, for the estimation of FSIQ and GAI scores. In the case of our sample, this outcome led us to question the usefulness of relying on a theory of cognitive aptitudes such as that of the CHC model, in order to determine the specific cognitive characteristics of IGC. We are of the opinion that the elaboration of a short form should take these specific cognitive characteristics into account, in order to obtain sufficiently accurate identification of IGC.

## Ethics statement

The participant's data were retrieved from clinical psychologists as part of their day-to-day clinical practice and a further examination ran with aim of developing a short-form of the Wechsler' scale.

## Author contributions

AA and BB designed the study. AA performed data collection and analyzed the data. AA and BB wrote the manuscript.

### Conflict of interest statement

The authors declare that the research was conducted in the absence of any commercial or financial relationships that could be construed as a potential conflict of interest.

## Supplementary material

Data Sheet 1 Raw data.

## References

[B1] Alloway T. P. Elsworth M. (2012). An investigation of cognitive skills and behavior in high ability students. Learn. Individ. Differ. 22, 891–895. 10.1016/j.lindif.2012.02.001

[B2] Assouline S. G. Foley-Nicpon M. Whiteman C. (2010). Cognitive and psychosocial characteristics of gifted students with written language disability. Gifted Child Q. 54, 102–115. 10.1177/0016986209355974

[B3] Aubry A. Bourdin B. (2016). Les Tests BV9 et B53 Peuvent-ils Prédire la Réussite Scolaire? [Can the B53 and BV9 Tests Predict the Academic Achievement?]. L'orientation Scolaire et Professionnelle 45/3 | 2016.

[B4] Brasseur S. Grégoire J. (2010). L'intelligence émotionnelle – trait chez les adolescents à haut potentiel : spécificités et liens avec la réussite scolaire et les compétences sociales [Trait emotional intelligence in adolescents with high potential: specificities and links with academic achievement and social competences]. Enfance 2010, 59–76. 10.4074/S0013754510001060

[B5] Calero M. D. García-Martín M. B. Jiménez M. I. Kazén M. Araque A. (2007). Self-regulation advantage for high-IQ children: Findings from a research study. Learn. Individ. Differ. 17, 328–343. 10.1016/j.lindif.2007.03.012

[B6] Carman C. A. (2013). Comparing apples and oranges: fifteen years of definitions of giftedness in research. J. Adv. Acad. 24, 52–70. 10.1177/1932202X12472602

[B7] Cornoldi C. Giofrè D. Orsini A. Pezzuti L. (2014). Differences in the intellectual profile of children with intellectual vs. learning disability. Res. Dev. Disabil. 35, 2224–2230. 10.1016/j.ridd.2014.05.013 24927516

[B8] Crawford J. R. Anderson V. Rankin P. M. MacDonald J. (2010). An index-based short form of the WISC-IV with accompanying analysis of the reliability and abnormality of differences. Br. J. Clin. Psychol. 49, 235–258. 10.1348/014466509X455470 19646334

[B9] Cyr J. J. Brooker B. H. (1984). Use of appropriate formulas for selecting WAIS–R short forms. J. Consult. Clin. Psychol. 52, 903–905. 10.1037/0022-006X.52.5.903

[B10] Dasi C. Soler M. J. Bellver V. Ruiz J. C. (2014). Short form of Spanish version of the WISC-IV for intelligence assessment in elementary school children. Psychol. Rep. 115, 784–793. 10.2466/03.PR0.115c32z7 25539178

[B11] Denney D. A. Ringe W. K. Lacritz L. H. (2015). Dyadic short forms of the Wechsler Adult Intelligence Scale-IV. Arch. Clin. Neuropsychol. 30, 404–412. 10.1093/arclin/acv035 26058660

[B12] Diedenhofen B. Musch J. (2015). cocor: a Comprehensive Solution for the Statistical Comparison of Correlations. PLoS ONE 10:e121945. 10.1371/journal.pone.0121945 25835001 PMC4383486

[B13] Dirks J. Wessels K. Quarfoth J. Quenon B. (1980). Can short-form WISC-R IQ tests identify children with high full scale IQ? Psychol. Sch. 17, 40–46.

[B14] Donders J. Elzinga B. Kuipers D. Helder E. Crawford J. R. (2013). Development of an eight-subtest short form of the WISC-IV and evaluation of its clinical utility in children with traumatic brain injury. Child Neuropsychol. 19, 662–670. 10.1080/09297049.2012.723681 22950806

[B15] Doppelt J. E. (1956). Estimating the full scale score on the Wechsler Adult Intelligence Scale from scores on four subjects. J. Consult. Psychol. 20, 63–66. 10.1037/h0044293 13295435

[B16] Evers A. Muñiz J. Bartram D. Boben D. Egeland J. Fernández-Hermida J. R. (2012). Testing practices in the 21st century. Eur. Psychol. 17, 300–319. 10.1027/1016-9040/a000102

[B17] Fawcett T. (2006). An introduction to ROC analysis. Pattern Recognit. Lett. 27, 861–874. 10.1016/j.patrec.2005.10.010

[B18] Fiorello C. A. Hale J. B. McGrath M. Ryan K. Quinn S. (2001). IQ interpretation for children with flat and variable test profiles. Learn. Individ. Differ. 13, 115–125. 10.1016/S1041-6080(02)00075-4

[B19] Flanagan D. P. Kaufman A. S. (2009). Essentials of WISC-IV assessment. Hoboken, NJ: John Wiley & Sons, Inc.

[B20] Gamer M. Lemon J. Singh I. F. P. (2012). irr: Various Coefficients of Interrater Reliability and Agreement (Version 0.84) [Computer Software]. Available online at: https://cran.r-project.org/web/packages/irr/

[B21] Genz A. Bretz F. Miwa T. Mi X. Leisch F. Scheipl F. (2018). Multivariate Normal and t Distributions. Available online at: http://mvtnorm.r-forge.r-project.org

[B22] Gignac G. E. (2015). Raven's is not a pure measure of general intelligence: implications for g factor theory and the brief measurement of g. Intelligence 52, 71–79. 10.1016/j.intell.2015.07.006

[B23] Gignac G. E. Watkins M. W. (2013). Bifactor modeling and the estimation of model-based reliability in the WAIS-IV. Multivar. Behav. Res. 48, 639–662. 10.1080/00273171.2013.804398 26741057

[B24] Giofrè D. Toffalini E. Altoè G. Cornoldi C. (2017). Intelligence measures as diagnostic tools for children with specific learning disabilities. Intelligence 61, 140–145. 10.1016/j.intell.2017.01.014

[B25] Girard T. A. Axelrod B. N. Patel R. Crawford J. R. (2015). Wechsler adult intelligence scale-IV dyads for estimating global intelligence. Assessment 22, 441–448. 10.1177/1073191114551551 25271008

[B26] Girard T. A. Axelrod B. N. Wilkins L. K. (2010). Comparison of WAIS-III short forms for measuring index and full-scale scores. Assessment 17, 400–405. 10.1177/1073191110369763 20519737

[B27] Girard T. A. Christensen B. K. (2008). Clarifying problems and offering solutions for correlated error when assessing the validity of selected-subtest short forms. Psychol. Assess. 20, 76–80. 10.1037/1040-3590.20.1.76 18315402

[B28] Golay P. Reverte I. Rossier J. Favez N. Lecerf T. (2013). Further insights on the French WISC–IV factor structure through Bayesian structural equation modeling. Psychol. Assess. 25, 496–508. 10.1037/a0030676 23148651

[B29] Gonthier C. Aubry A. Bourdin B. (2017). Measuring working memory capacity in children using adaptive tasks: example validation of an adaptive complex span. Behav. Res. Methods 27, 1–12. 10.3758/s13428-017-0916-4 28643158

[B30] Hale J. B. Fiorello C. A. Kavanagh J. A. Holdnack J. A. Aloe A. M. (2007). Is the demise of IQ interpretation justified? A response to special issue authors. Appl. Neuropsychol. 14, 37–51. 10.1080/09084280701280445

[B31] Hoard M. K. Geary D. C. Byrd-Craven J. Nugent L. (2008). Mathematical cognition in intellectually precocious first graders. Dev. Neuropsychol. 33, 251–276. 10.1080/87565640801982338 18473199

[B32] Hrabok M. Brooks B. L. Fay-McClymont T. B. Sherman E. M. S. (2012). Wechsler Intelligence Scale for Children-Fourth Edition (WISC-IV) short-form validity: a comparison study in pediatric epilepsy. Child Neuropsychol. 20, 49–59. 10.1080/09297049.2012.741225 23216421

[B33] Karnes F. A. Brown K. E. (1981). A short form of the WISC-R for gifted students. Psychol. Schools 18, 169–173.

[B34] Keith T. Z. Fine J. G. Taub G. E. Reynolds M. R. Kranzler J. H. (2006). Higher order, multisample, confirmatory factor analysis of the wechsler intelligence scale for children - fourth edition: what does it measure? in School Psychology Review 35, 108–127. Available online at: http://eric.ed.gov/?id=EJ788234

[B35] Kelley K. (2018). MBESS (Version 4.4.3) [Computer Software]. Available online at: https://cran.r-project.org/package=MBESS

[B36] Kelley K. Pornprasertmanit S. (2016). Confidence intervals for population reliability coefficients: evaluation of methods, recommendations, and software for composite measures. Psychol. Methods 21, 69–92. 10.1037/a0040086 26962759

[B37] Kieng S. Rossier J. Favez N. Lecerf T. (2013). Étude exploratoire de la stabilité à long terme des indices standard du WISC-IV [Exploratory study about long-term stability of French WISC-IV index scores]. Pratiques Psychologiques 19, 163–178. 10.1016/j.prps.2013.07.003

[B38] Killan J. B. Hughes L. C. (1978). A comparison of short forms of the intelligence scale for children - revised in the screening of gifted referrals. Gifted Child Q. 22, 111–115. 10.1177/001698627802200123

[B39] Kroesbergen E. H. van Hooijdonk M. van Viersen S. Middel-Lalleman M. M. N. Reijnders J. J. W. (2016). The psychological well-being of early identified gifted children. Gifted Child Q. 60, 16–30. 10.1177/0016986215609113

[B40] Lakens D. (2013). Calculating and reporting effect sizes to facilitate cumulative science: a practical primer for t-tests and ANOVAs. Front. Psychol. 4:863. 10.3389/fpsyg.2013.00863 24324449 PMC3840331

[B41] Lecerf T. Bovet-Boone F. Peiffer E. Kieng S. Geistlich S. (2016). WISC-IV GAI and CPI profiles in healthy children and children with learning disabilities. Rev. Eur. Psychol. Appl. 66, 101–107. 10.1016/j.erap.2016.04.001

[B42] Lecerf T. Kieng S. Geistlich S. (2015). Cohésion–non-cohésion des scores composites : valeurs seuils et interprétabilité. L'exemple du WISC-IV [Cohesive vs. non cohesive composite scores: Cut-off values and interpretability. The example of the WISC-IV]. Pratiques Psychologiques 21, 155–171. 10.1016/j.prps.2015.02.001

[B43] Lecerf T. Reverte I. Coleaux L. Favez N. Rossier J. (2010a). Indice d'Aptitude Général pour le WISC-IV: Normes francophones [General ability index for the WISC-IV: French norms]. Pratiques Psychologiques 16, 109–121. 10.1016/j.prps.2009.04.001

[B44] Lecerf T. Rossier J. Favez N. Reverte I. Coleaux L. (2010b). The Four- vs. Alternative Six-Factor Structure of the French WISC-IV. Swiss J. Psychol. 69, 221–232. 10.1024/1421-0185/a000026

[B45] Levy P. (1967). The correction for spurious correlation in the evaluation of short-form tests. J. Clin. Psychol. 23, 84–86. 6031038 10.1002/1097-4679(196701)23:1<84::aid-jclp2270230123>3.0.co;2-2

[B46] Levy P. (1968). Short-form tests: a methodological review. Psychol. Bull. 69, 410–416. 10.1037/h0025736 4873059

[B47] Litster K. Roberts J. (2011). The self-concepts and perceived competencies of gifted and non-gifted students: a meta-analysis. J. Res. Special Educ. Needs 11, 130–140. 10.1111/j.1471-3802.2010.01166.x

[B48] Lovett B. J. Lewandowski L. J. (2006). Gifted students with learning disabilities: who are they? J. Learn. Disabil. 39, 515–527. 10.1177/00222194060390060401 17165619

[B49] Maehler C. Schuchardt K. (2009). Working memory functioning in children with learning disabilities: does intelligence make a difference? J. Intellect. Disabil. Res. 53, 3–10. 10.1111/j.1365-2788.2008.01105.x 19093981

[B50] Margulies A. S. Floyd R. G. (2009). A Preliminary Examination of the CHC Cognitive Ability Profiles of Children with High IQ and High Academic Achievement Enrolled in Services for Intellectual Giftedness. Nashville, TN: Woodcock Munoz Foundation Press.

[B51] Mark R. Beal A. L. Dumont R. (1998). Validation of a WISC-III Short-Form for the Identification of Canadian Gifted Students. Can. J. School Psychol. 14, 1–10. 10.1177/082957359801400101

[B52] McBee M. T. Peters S. J. Miller E. M. (2016). The impact of the nomination stage on gifted program identification a comprehensive psychometric analysis. Gifted Child Q. 60, 258–278. 10.1177/0016986216656256

[B53] McBee M. T. Peters S. J. Waterman C. (2013). Combining scores in multiple-criteria assessment systems: the impact of combination rule. Gifted Child Q. 58, 69–89. 10.1177/0016986213513794

[B54] McClain M.-C. Pfeiffer S. (2012). Identification of gifted students in the united states today: a look at state definitions, policies, and practices. J. Appl. School Psychol. 28, 59–88. 10.1080/15377903.2012.643757

[B55] McGraw K. O. Wong S. P. (1996). Forming inferences about some intraclass correlation coefficients. Psychol. Methods 1, 30–46. 10.1037/1082-989X.1.1.30

[B56] McIntosh D. E. Dixon F. A. Pierson É. E. (2005). “Use of intelligence tests in the identification of giftedness,” in Contemporary Intellectual Assessment, eds Harrison P. L. Flanagan D. P., 2nd Edn (New York, NY: Guilford Press), 504–520.

[B57] McKenzie K. Murray A. L. Murray K. R. Murray G. C. (2013). Assessing the accuracy of the WISC-IV seven-subtest short form and the child and adolescent intellectual disability screening questionnaire in identifying intellectual disability in children. Child Neuropsychol. 20, 372–377. 10.1080/09297049.2013.799642 23745760

[B58] Meyers J. E. Zellinger M. M. Kockler T. Wagner M. Miller R. M. (2013). A validated seven-subtest short form for the WAIS-IV. Appl. Neuropsychol Adult 20, 249–256. 10.1080/09084282.2012.710180 23530602

[B59] Murray A. L. McKenzie K. Murray G. C. (2016). An evaluation of the performance of the WISC-IV eight-subtest short form with children who may have an intellectual disability. J. Intellect. Dev. Disabil. 41, 50–53. 10.3109/13668250.2015.1084611

[B60] Neihart M. (1999). The impact of giftedness on psychological well-being: What does the empirical literature say? Roeper Rev. 22, 10–17. 10.1080/02783199909553991

[B61] Newman T. M. Sparrow S. S. Pfeiffer S. I. (2008). “The use of the WISC-IV in assessment and intervention planning for children who are gifted,” in WISC-IV. Clinical Assessment and Intervention, eds Prifitera A. Saklofske D. H. Weiss L. G. (San Diego, CA: Academic Press), 217–242.

[B62] Newton J. H. McIntosh D. E. Dixon F. Williams T. Youman E. (2008). Assessing giftedness in children: comparing the accuracy of three shortened measures of Intelligence to the Stanford–Binet Intelligence Scales, Fifth Edition. Psychol. Sch. 45, 523–536. 10.1002/pits.20321

[B63] Ortiz V. Z. Gonzalez A. (1989). Validation of a short form of the WISC-R with accelerated and gifted hispanic students. Gifted Child Q. 33, 152–155. 10.1177/001698628903300405

[B64] Pfeiffer S. I. Jarosewich T. (2003). Gifted Rating Scales San Antonio, TX: Pearson.

[B65] Pierson É. E. Kilmer L. M. Rothlisberg B. A. McIntosh D. E. (2012). Use of brief intelligence tests in the identification of giftedness. J. Psychoeduc. Assess. 30, 10–24. 10.1177/0734282911428193

[B66] Prewett P. N. (1995). A comparison of two screening tests (the Matrix Analogies Test—Short Form and the Kaufman Brief Intelligence Test) with the WISC-III. Psychol. Assess. I, 69–72. 10.1037/1040-3590.7.1.69

[B67] R Core Team (2017). R: A Language and Environment for Statistical Computing. Vienna: R Foundation for Statistical Computing Available online at: https://www.R-project.org/

[B68] Reise S. P. Bonifay W. E. Haviland M. G. (2013). Scoring and modeling psychological measures in the presence of multidimensionality. J. Pers. Assess. 95, 129–140. 10.1080/00223891.2012.725437 23030794

[B69] Reiter B. A. (2004). The Application of a WISC-III Short Form for Screening Gifted Elementary Students in Canada. Can. J. School Psychol. 19, 191–203. 10.1177/082957350401900110

[B70] Reverte I. Golay P. Favez N. Rossier J. Lecerf T. (2015). Testing for multigroup invariance of the WISC-IV structure across France and Switzerland: Standard and CHC models. Learn. Individ. Differ. 40, 127–133. 10.1016/j.lindif.2015.03.015

[B71] Reynolds M. R. Keith T. Z. (2017). Multi-group and hierarchical confirmatory factor analysis of the Wechsler Intelligence Scale for Children—Fifth Edition: What does it measure? Intelligence 62, 31–47. 10.1016/j.intell.2017.02.005

[B72] Rinn A. N. Bishop J. (2015). Gifted adults: a systematic review and analysis of the literature. Gifted Child Q. 59, 213–235. 10.1177/0016986215600795

[B73] Robin X. Turck N. Hainard A. Tiberti N. Lisacek F. Sanchez J.-C.. (2011). pROC: an open-source package for R and S+ to analyze and compare ROC curves. BMC Bioinformatics 12:77. 10.1186/1471-2105-12-77 21414208 PMC3068975

[B74] Rowe E. W. Kingsley J. M. Thompson D. F. (2010). Predictive ability of the General Ability Index (GAI) versus the Full Scale IQ among gifted referrals. School Psychol. Q. 25, 119–128. 10.1037/a0020148

[B75] Ruthsatz J. Urbach J. B. (2012). Child prodigy: a novel cognitive profile places elevated general intelligence, exceptional working memory and attention to detail at the root of prodigiousness. Intelligence 40, 419–426. 10.1016/j.intell.2012.06.002

[B76] Ryan J. J. Glass L. A. Brown C. N. (2007). Administration time estimates for Wechsler Intelligence Scale for Children-IV subtests, composites, and short forms. J. Clin. Psychol. 63, 309–318. 10.1002/jclp.20343 17211870

[B77] Ryan J. J. Ward L. C. (1999). Validity, reliability, and standard errors of measurement for two seven-subtest short forms of the Wechsler Adult Intelligence Scale—III. Psychol. Assess. 11, 207–211. 10.1037/1040-3590.11.2.207

[B78] Saklofske D. H. Weiss L. G. Raiford S. E. Prifitera A. (2005). “Advanced interpretive issues with the WISC-IV Full-Scale IQ and General Ability Index Scores,” in WISC-IV Advanced Clinical Interpretation, eds Weiss L. G. Saklofske D. H. Prifitera A. Holdnack J. (San Diego: Elsevier Academic Press), 99–138.

[B79] Sattler J. M. Ryan J. J. (2009). Assessment with the WAIS-IV. La Mesa, CA: Jerome M. Sattler, Publisher, Inc.

[B80] Shaw P. Greenstein D. Lerch J. Clasen L. Lenroot R. Gogtay N.. (2006). Intellectual ability and cortical development in children and adolescents. Nature 440, 676–679. 10.1038/nature04513 16572172

[B81] Shi J. Tao T. Chen W. Cheng L. Wang L. Zhang X. (2013). Sustained attention in intellectually gifted children assessed using a continuous performance test. PLoS ONE 8:e0057417. 10.1371/journal.pone.0057417 23451224 PMC3581460

[B82] Silverstein A. B. (1990). Short forms of individual intelligence tests. Psychol. Assess. 2, 3–11. 10.1037/1040-3590.2.1.3

[B83] Simpson M. Carone D. A. Burns W. J. Seidman T. Montgomery D. Sellers A. (2002). Assessing giftedness with the WISC-III and the SB-IV. Psychol. Sch. 39, 515–524. 10.1002/pits.10057

[B84] Spearman C. (1904). “General Intelligence,” Objectively Determined and Measured. Am. J. Psychol. 15, 201–293.

[B85] Tellegen A. Briggs P. F. (1967). Old wine in new skins: grouping wechsler subtests into new scales. J. Consult. Psychol. 31, 499–506. 10.1037/h0024963 6075979

[B86] Thomeer M. L. Lopata C. Volker M. A. Toomey J. A. Lee G. K. Smerbeck A. M. (2012). Randomized clinical trial replication of a psychosocial treatment for children with high-functioning autism spectrum disorders. Psychol. Sch. 49, 942–954. 10.1002/pits.21647

[B87] Toffalini E. Giofrè D. Cornoldi C. (2017a). Strengths and weaknesses in the intellectual profile of different subtypes of specific learning disorder: a study on 1,049 diagnosed children. Clin. Psychol. Sci. 5, 402–409. 10.1177/2167702616672038

[B88] Toffalini E. Pezzuti L. Cornoldi C. (2017b). Einstein and dyslexia: Is giftedness more frequent in children with a specific learning disorder than in typically developing children? Intelligence 62, 175–179. 10.1016/j.intell.2017.04.006

[B89] van Viersen S. de Bree E. H. Kroesbergen E. H. Slot E. M. de Jong P. F. (2015). Risk and protective factors in gifted children with dyslexia. Ann. Dyslexia 65, 178–198. 10.1007/s11881-015-0106-y 26269395 PMC4565890

[B90] van Viersen S. Kroesbergen E. H. Slot E. M. de Bree E. H. (2014). High reading skills mask dyslexia in gifted children. J. Learn. Disabil. 49, 189–199. 10.1177/0022219414538517 24935885

[B91] Volker M. A. Lopata C. Cook-Cottone C. (2006). Assessment of children with intellectual giftedness and reading disabilities. Psychol. Sch. 43, 855–869. 10.1002/pits.20193

[B92] Warne R. T. (2016). Five reasons to put the g back into giftedness: an argument for applying the cattell-horn-carroll theory of intelligence to gifted education research and practice. Gifted Child Q. 60, 3–15. 10.1177/0016986215605360

[B93] Watkins M. W. (2017). The reliability of multidimensional neuropsychological measures: from alpha to omega. Clin. Neuropsychol. 31, 1113–1126. 10.1080/13854046.2017.1317364 28429633

[B94] Watkins M. W. Greenawalt C. G. Marcell C. M. (2002). Factor Structure of the Wechsler Intelligence Scale for Children-Third Edition among Gifted Students. Educ. Psychol. Meas. 62, 164–172. 10.1177/0013164402062001011

[B95] Watkins M. W. Smith L. G. (2013). Long-term stability of the Wechsler Intelligence Scale for Children–Fourth Edition. Psychol. Assess. 25, 477–483. 10.1037/a0031653 23397927

[B96] Wechsler D. (2005). Manuel de l'Echelle d'Intelligence de Wechsler pour Enfants – 4e édition [Manual for the Wechsler Intelligence Scale for Children – fourth edition]. Paris: Editions du Centre de Psychologie Appliquée.

[B97] Wechsler D. (2016). Manuel de l'Echelle d'Intelligence de Wechsler pour Enfants 5e édition [Manual for the Wechsler Intelligence Scale for Children – fifth edition]. Paris: Editions du Centre de Psychologie Appliquée.

[B98] Weiss L. G. Beal A. L. Saklofske D. H. Alloway T. P. Prifitera A. (2008). “Interpretation and intervention with WISC-IV in the clinical assessment context,” in WISC-IV. Clinical Assessment and Intervention, eds Prifitera A. Saklofske D. H. Weiss L. G. (San Diego: Elsevier Inc), 3–66.

[B99] Weiss L. G. Keith T. Z. Zhu J. Chen H. (2013). WISC-IV and Clinical validation of the four- and five-factor interpretative approaches. J. Psychoeduc. Assess. 31, 114–131. 10.1177/0734282913478032

[B100] Winner E. (2000). The origins and ends of giftedness. Am. Psychol. 55, 159–169. 10.1037/0003-066X.55.1.159 11392860

[B101] Zou G. Y. (2007). Toward using confidence intervals to compare correlations. Psychol. Methods 12, 399–413. 10.1037/1082-989X.12.4.399 18179351

